# Effect of extrusion of soybean meal on feed spectroscopic molecular structures and on performance, blood metabolites and nutrient digestibility of Holstein dairy calves

**DOI:** 10.5713/ajas.19.0899

**Published:** 2020-03-04

**Authors:** Ammar Mollaei Berenti, Mojtaba Yari, Saeed Khalaji, Mahdi Hedayati, Amin Akbarian, Peiqiang Yu

**Affiliations:** 1 Department of Animal Science, College of Agriculture, Malayer University, 65719-95863, Malayer, Iran; 2 Nardaneh Faravar Feedar Company, Kharazmi Industrial Park, Sharif Abad, Pak Dasht, Tehran, 33931-99008, Iran; 3 Department of Animal and Poultry Science, University of Saskatchewan, Saskatoon S7N 5A8, Canada

**Keywords:** Amides, Calves, Performance, Protein Digestibility, Rumen

## Abstract

**Objective:**

Performance and physiological responses of dairy calves may change by using extruded soybean meal (ESBM) instead of common soybean meal (SBM) in starter feed. The aims of the current study were i) to determine the effect of extrusion processing of SBM on protein electrophoretic size, fourier transform infrared spectroscopy (FTIR) structures and Cornell Net Carbohydrate and Protein System (CNCPS) protein sub-fractions and ii) to determine the effect of substitution of SBM with ESBM in starter feed of Holstein heifer calves during pre and post-weaning on performance, nutrient digestibility, and blood metabolites.

**Methods:**

The SBM was substituted with ESBM at the level of 0%, 25%, 50%, 75%, and 100% (dry matter [DM] basis). Fifty heifer calves (initial body weight 40.3±0.63 kg) were used for the study. After birth, animals were fed colostrum for 3 days and then they were fed whole milk until weaning. Animals had free access to starter feed and water during the study.

**Results:**

Extrusion of SBM decreased electrophoretic protein size and increased rapidly degradable true protein fraction, changed FTIR protein and amide II region. With increasing level of ESBM in the diet, starter intake increased quadratically during the pre-weaning period (p<0.05) and body weight, DM intake and average daily gain increased linearly during the post-weaning and the whole study period (p<0.05). Tbe DM and crude protein digestibilities at week 14 and blood glucose and beta hydroxybutyric acid increased linearly in calves as the level of ESBM increased in the diet (p<0.05).

**Conclusion:**

Dairy calves performance and physiological responses were sensitive to SBM protein characteristics including electrophoretic size, FTIR structures and CNCPS protein fractions.

## INTRODUCTION

In bio-polymers exposed to the extrusion cooking, physico-chemical changes such as losses of original conformation, breaking and binding and thermal degradation might happen. Proteins are the most sensitive nutrients to the such conditions because they contains a large number of highly reactive functional groups [1]. Protein denaturation is a consequence of extrusion process which would result to increased protein availability for gastrointestinal enzymes, decreased the solubility of protein, and deactivation of anti-nutritional factors [2]. Therefore extrusion cooking condition may changes molecular structures, weight and size of proteins which these changes may be resulted to changes in crude protein (CP) sub-fractions. Also, these changes may affect the palatability and overall the feeding value of final product for a ruminant [1,2].

Soybean meal (SBM) is the most important protein source used in starter feed for dairy calves because of its desirable amino acid balance, high digestibility and its palatability. The SBM contains several anti-nutritional factors, however, which could reduce nutrient availability and growth performance of calves [3,4].

Different forms of SBM have been used in starter feed for dairy calves including unprocessed [5], extruded [6], fermented [7], xylose treated [8] or heat treated [9]. Heat treatment is the most common, and probably the most feasible processing method, for feedstuffs such as SBM. Temperature and duration of heating must be carefully controlled with feeds high in protein to optimize the content of digestible protein and to prevent enhancement of the undigestible protein fraction or other heat damage [5].

To date, there is little information on the releation between performance and physiological responses of dairy calves during pre and post-weaning to SBM protein charactristics. The objectives of the current study were, i) to study the effect of extrusion processing of SBM on its protein fourier transform infrared spectroscopy (FTIR) molecular structures, protein fractions and protein electrophoretic weight and size, and ii) to determine the effect of substitution of SBM with extruded SBM (ESBM) in ground starter feed on growth performance, dry matter intake (DMI), body size measurements, blood metabolites and total tract apparent nutrient digestibility during the pre and post-weaning period in Holstein calves.

## MATERIALS AND METHODS

### Extrusion processing

A batch of the SBM separated into 2 equal portions, with the first portion being used as a control SBM source. The second portion of SBM was ESBM at a high temperature (150°C± 2°C) along with moisture (25% to 30%) and at a high pressure for 15 s by a single screw (speed of 450 rpm, diameter of 10 cm) double conditioner extruder system (Amandus Kahl, Expander, OEE 32, GmbH and Co., KG, Reinbek, Germany) at Yasna Mehr Co. (Tehran, Iran) according to Jahanian and Rasouli [10]. Sub-samples of SBM (n = 5) and ESBM (n = 5) were collected for analysis of chemical composition, CP fractionation (according to Cornell Net Carbohydrate and Protein System [CNCPS]), FTIR molecular structures and gel electrophoresis.

### Chemical composition, protein fractionation and sodium dodecyl sulphate-polyacrylamide gel electrophoresis

**Figure 1:**
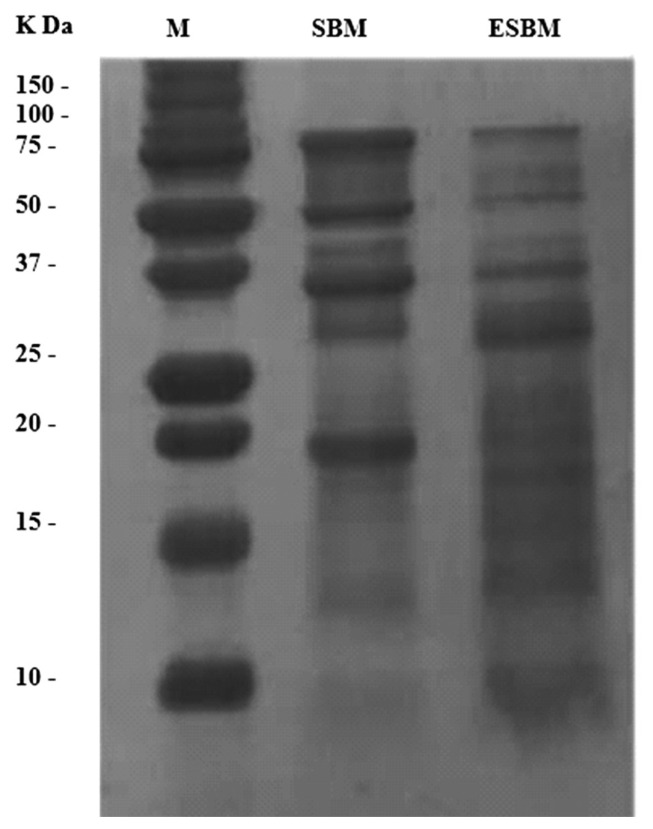
Sodium dodecylsulphate-polyacrylamide gel electrophoresis (SDS-PAGE) pattern of protein marker (M), soybean meal (SBM) and extruded soybean meal (ESBM) samples.

The SBM and ESBM sub-samples were oven dried at 50°C for 48 h, ground through a 1-mm screen and then stored in a freezer at −20°C until analyses. Dry matter (DM), crude ash, CP, and ether extract (EE) were determined according to the standard procedures of the AOAC [11]. Neutral detergent fiber and acid detergent fiber were determined according to the method described by Van Soest et al [12]. Neutral detergent insoluble CP (NDICP) and acid detergent insoluble CP were determined as described by Licitra et al [13]. The CNCPS system was used to divide CP into five fractions with different degradation characteristics [13]. Protein molecular weight distribution of SBM and ESBM measured by a sodium dodecyl sulphate-polyacrylamide gel electrophoresis (SDS-PAGE) dis-continuous system as described by Sadeghi et al [2]. The sub-units of the gel was determined by densitometric scanning at 580 nm (Figure 1).

### Molecular spectroscopic study

**Figure 2:**
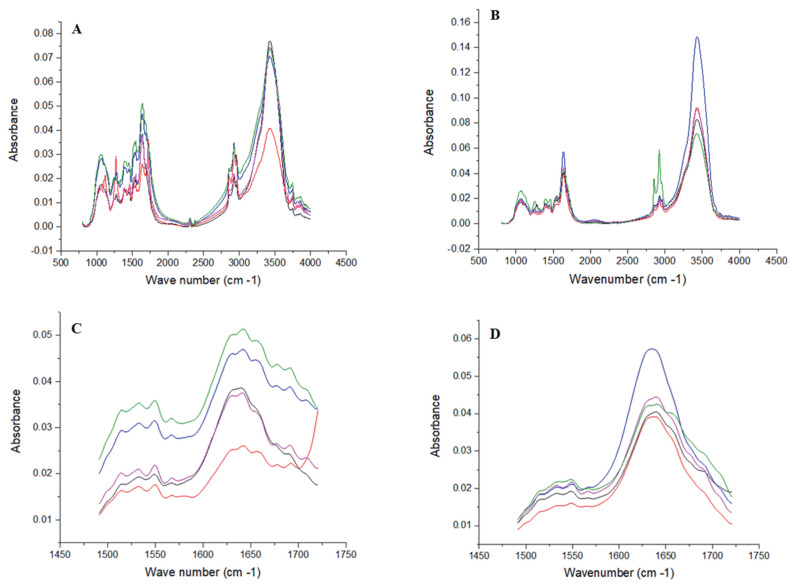
Full fourier transform infrared spectroscopy (FTIR) spectrum from region 800 to 4,000 cm^−1^ (A and B) and protein area (ca. 1,720 to 1,485 cm^−1^; C and D) respectively of soybean meal and extruded soybean meal samples (n = 5).

The infrared (IR) absorbance band of samples was determined using a FTIR spectroscopy (Bruker Tensor 27, Bruker Optics Inc., Billerica, MA, USA) coupled with a universal attenuated total reflectance accessory. The sub-samples were finely ground and pressed uniformly against the diamond surface using a spring-loaded anvil, and the mid-IR spectra recorded from a resolution of 800 to 4,000 cm^−1^ at 2 cm^−1^. Each sub-sample was scanned twice and averaged before further analysis. The collected spectra were corrected against air as background. The full FTIR spectrum and protein region of different sub-samples are shown in Figure 2.

### Spectral analysis

The baselines were corrected and the data were normalized with Origin Pro (OriginLab, Northampton, MA, USA). The parameters in terms of amide I and II peak area and height, alpha-helix and beta-sheet peak height, and their ratios, total carbohydrates and non-structural carbohydrates were determined according to published reports [14,15]. In this study, the analyzed spectral baseline for protein was *ca*. 1,720 to 1,485 cm^−1^; the spectral regions for amide I and II were *ca*. 1,720 to 1,575, and 1,575 to 1,485 cm^−1^, respectively. Typical multi-component peak fitting of a typical second derivative spectrum in the protein amide I region of different treatments by Lorentzian and Gause model was performed to find protein molecular secondary structures including alpha-helices, beta-sheets and others. For the alpha-helix and beta-sheets, the peak fell within the range of *ca*. 1,650 to 1,660 and 1,620 to 1,640 cm^−1^, respectively. Different ratios were calculated according to respective absorbance intensity value. Total carbohydrates at *ca*. 800 to 1,185 cm^−1^ and non-structural carbohydrates at *ca*. 950 to 1,065 cm^−1^ were also determined [14,15].

### Multivariate analysis

**Figure 3:**
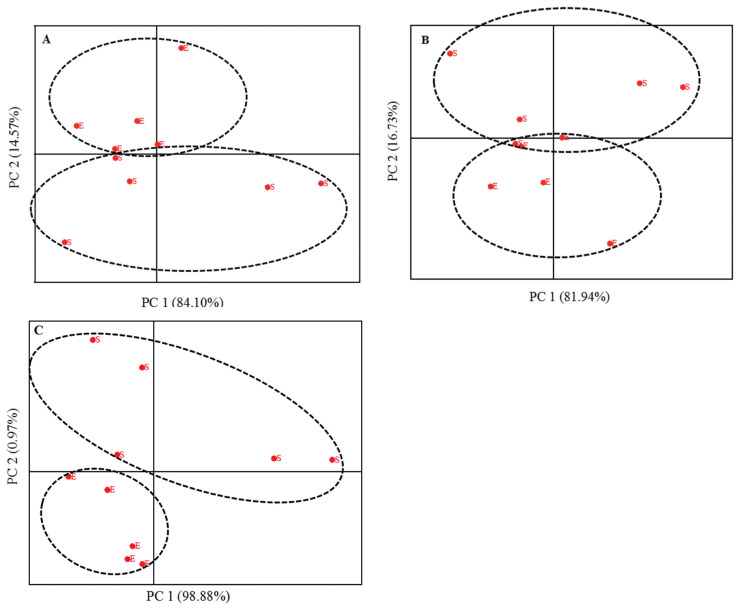
Principle component analysis of protein area (A; ca. 1,720 to 1,485 cm^−1^), amide I area (B; ca. 1,720 to 1,575 cm^−1^) and of amide II area (C; ca. 1,575 to 1,485 cm^−1^) of soybean meal (code S) and extruded soybean meal (code E) samples (n = 5).

**Figure 4:**
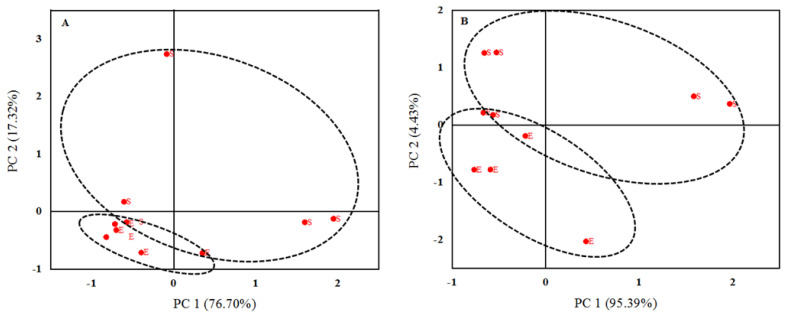
Principle component analysis of total carbohydrates area (A; ca. 800 to 1,185 cm^−1^) and non-structural carbohydrates area (B; ca. 950 to 1,065 cm^−1^) of soybean meal (code S) and extruded soybean meal (code E) samples (n = 5).

The FTIR region of protein (Figure 3A), amide I (Figure 3B), amide II (Figure 3C), total carbohydrates (Figure 4A), and non-structural carbohydrates (Figure 4B) of sub-samples were analyzed using principal component analysis (PCA) by Origin software (OriginPro, OriginLab, USA) [15]. These analyses classify and discriminate inherent structural differences, and then detect the main sources of variation within the protein, amide I, amide II, total carbohydrates and non-structural carbohydrates finger print spectra.

### Calves, management and diets

The animal trial was carried out at Dasht-e-Novin Dairy Farm, Malayer, Iran, according to the guidelines of the Iranian Council of Animal Care [16]. Fifty Holstein calves with initial body weight of 40.3±0.63 kg were used. After birth, animals were fed colostrum from their dams; 2 to 2.5 L of colostrum during the first three feedings (i.e., within 1.5 h after birth and at 3 and 6 hour after the first feeding). Colostrum feeding was continued for another 3 days at 2 L per meal and three meals per day.

Animals were randomly assigned to 5 dietary treatments (n = 10 per treatment) in a completely randomized design arrangement. Treatments were the substitution of SBM with ESBM at the level of 0% (SBM), 25% (ESBM25), 50% (ESBM 50), 75% (ESBM75), and 100% (ESBM100) in starter feed (Table 1). Beginning at day 4 of life, animals had free access to starter and drinking water. Calves were housed in individual pens (1.2×2.5 m) bedded with chopped straw, which was renewed every 24 h. Calves were fed whole milk in galvanized tin buckets twice a day at 0700 and 1800 h. Calves received 4 L/d from day 3 to 21, 7 L/d from day 21 to 50, 4 L/d from day 50 to d 60 and 2 L/d (once a day) from day 60 to 70 of age. Calves were weaned at 70 d of age, but remained in their individual pens and were fed their respective starter feed *ad-libitum* (at least 5% orts) until 21 days after weaning. Starter feed intake was recorded daily during the whole trial.

### Sample collection and chemical composition analysis

Fecal spot-samples were collected from 5 randomly selected calves from each treatment group during three periods; days 27, 28, and 29; days 68, 69, and 70; and days 89, 90, and 91. Feed samples were also collected at those days. Fecal and feed samples of the three days in each period were pooled per animal. These samples were oven dried at 50°C for 48 h and stored in a freezer at −20°C until chemical analysis. Acid insoluble ash was determined in collected feed and fecal samples and used as an internal marker to estimate apparent total tract digestibility of DM, organic matter (OM), and CP [17].

### Blood sample collection and analysis

Blood samples were collected from 5 randomly selected calves within each treatment group on days 30, 60, and 90 of the study. Blood samples were collected from the jugular vein into the10-mL tubes at 2 h after morning feeding and immediately placed on ice and transferred to laboratory. Samples were centrifuged at 3,000×*g* for 15 min at 4°C and serum was collected for further analysis. Serum metabolites including glucose, urea nitrogen, albumin, and total protein were determined spectrophotometrically (UNICCO, 2100; Zistchemi, Tehran, Iran) using commercial kits (Pars Azmoon Co., Tehran, Iran). The beta hydroxybutyric acid (BHBA) concentration was measured in whole blood using a commercial kit FreeStyle Optium H BHBA Test Strips).

### Body weight and size measurements and fecal scoring

Body weight, heart girth (circumference of the chest in cm), wither height (distance from the base of the front feet to wither in cm) were taken at the beginning of the experiment (day 3) and then at weekly intervals until the end of experimental period [9]. Fecal scores of all calves were recorded daily on a scale of 1 to 5, with 1 being normal, 2 being soft to loose, 3 being loose to watery, 4 being watery, mucous and slightly bloody, and 5 being watery, mucous and bloody [18]. Fecal scores for a calf during a week were averaged before statistical analysis.

### Statistical analysis

All data were analyzed by mixed procedure of SAS [19] in a complete randomized design using following models:

$$\begin{array}{l} {\text{Y}}_{\text{ijkl}}=\mu +{\text{T}}_{\text{i}}+{\text{W}}_{\text{j}}+{\text{C}}_{\text{k}}+{\text{F}}_{\text{l}}+{\text{T}}_{\text{i}}\times {\text{W}}_{\text{j}}+{\text{e}}_{\text{ijkl}}\\ {\text{Y}}_{\text{ijk}}=\mu +{\text{T}}_{\text{i}}+{\text{C}}_{\text{k}}+{\text{F}}_{\text{l}}+{\text{e}}_{\text{ikl}}\\ {\text{Y}}_{\text{ij}}=\mu +{\text{T}}_{\text{i}}+{\text{B}}_{\text{j}}+{\text{e}}_{\text{ij}} \end{array}$$

Where Y_ijkl_ is the observation of dependent variable; μ is the fixed effect of population mean for the variable; T_i_ is the fixed effect of treatment (i = 5; levels of SBM substitution with ESBM); W_j_ is the fixed effect of week (j = 10 for pre-weaning measurements and j = 3 for post-weaning measurements); C_k_ is the random effect of calf within treatment (k = 10 for each treatment); F_l_ is the covariate effect of the first measurements for body weight, heart girth and height; T_i_×W_j_ is the interaction between factor T at level i and factor W at level j, B_j_ is the random effect of block (j = 5 sub-samples from each SBM and ESBM) and e (e_ijkl_, e_ikl_, and e_ij_) is the random error associated to related observation. Model 1 was used for weekly measured traits including body weight gain, body heart girth and height, fecal score and starter intake during pre and post-weaning and the total period. Model 2 was used for first body weight, heart girth and height and weaning weight, final body weight, nutrient digestibility and blood analysis during post-weaning. Initial body weight, heart girth and body height measured at day 3 before the beginning of the experiment were included in the model as covariates to improve the precision of analysis. The covariate was excluded from the model when it was not significant (p>0.10). The effect of extrusion processing on chemical composition, protein fractions and FTIR molecular structures were analyzed using model 3. The analysis of treatment levels of linear, quadratic, and higher order effects were performed using polynomial orthogonal contrasts by the command CONTRAST in SAS. The adjust Tukey test was used for multiple treatments comparisons using the LSMEAN statement of SAS 9.2 [19]. For the different statistical tests, significance was declared at p≤0.05 and trend at p≤0.10.

## RESULTS

### Effect of soybean meal extrusion on nutrient profile, electrophoretic protein distribution and FTIR molecular structures

Extruded SBM had greater EE and rapidly degradable true protein fraction (PB1) and lesser non-protein nitrogen (PA) fraction compared with SBM (p<0.05; Table 2). Extrusion decreased protein sub-unites molecular weight of SBM as determined by SDS-PAGE (Figure 1). The FTIR molecular structures, analyzed by analysis of variance, were similar for SBM and ESBM, except for the amide I:amide II ratio, which was greater in ESBM (p<0.01; Table 2). Differences in FTIR inherent molecular structures between SBM and ESBM were fully discriminated at the protein region (Figure 3A), amide II region (Figure 3D) and non-structural carbohydrates region (Figure 4B) using PCA.

### Performance and nutrient digestibility of dairy calves

During pre-weaning period, weekly body weight increased linearly and quadratically with increasing ESBM level in the diet (p<0.01; Table 3). Weaning body weight increased linearly with increasing ESBM level in the diet (p<0.05). Starter DMI and total DMI tended to decrease (p = 0.054) quadratically and average daily gain (ADG) tended to increase linearly during pre-weaning with increasing ESBM level in the diet (p<0.10; Table 3).

During post-weaning, DMI and ADG increased as the level of ESBM increased in diet (p<0.10; Table 3) and feed conversion ratio (FCR) remained similar among treatments. Animals fed the highest level of ESBM had a greater weekly body weight than animals fed control and ESBM25, with those fed ESBM50 and ESBM75 intermediate (p<0.01).

Over the whole study, DMI and ADG increased linearly with increasing ESBM level in the diet (p<0.05), while FCR remained similar among treatments. Animals fed ESBM100 had a greater DMI and ADG than animals fed ESBM25, with other animals intermediate (p<0.05; Table 3). Body weight of animals increased linearly and quadratically with increasing ESBM level in the diet (p<0.01).

At week 4, apparent digestibility of DM, OM, and CP changed in a cubic pattern with increasing ESBM level in the diet (Table 4). At week 10, DM and CP digestibilities tended to change in a cubic pattern as the level of ESBM in diet increased (Table 4; p = 0.08). At week 13, DM, OM, and CP digestibilities increased linearly (tendency for OM digesibility) as the level of ESBM in diet increased (p<0.05). Animals fed ESBM100 had a greater CP digestibility compared with those fed control and ESBM50 diets, with those fed ESBM25 and ESBM75 intermediate (p<0.01; Table 4).

### Body size measurements

Pre-weaning, wither height of animals increased linearly with increasing ESBM level in the diet (p<0.05; Table 5). Wither height was greater in animals fed ESBM100 than those fed ESBM25, with those fed the other three diets having intermediate values (p<0.05). Hearth girth increased linearly as the level of ESBM increased with those fed ESBM100 having the largest hearth girth (p<0.01).

Post-weaning, hearth girth of animals increased linearly and quadratically with increasing ESBM level in the starter feed (p<0.05). Animals fed ESBM100 having a larger hearth girth than those fed ESBM25, with those fed other dietsintermediate (p<0.05; Table 5).

Over the whole experiment, wither height of animals increased linearly and quadratically as the level of ESBM in the starter increased (p<0.05). Animals fed ESBM100 starter had a greater wither height than those fed ESBM25, with those fed the other diets intermediate (p<0.05). Hearth girth of animals increased linearly and quadratically as the level of ESBM in the starter increased (p<0.01), with those fed ESBM25 and ESBM50 having a smaller hearth girth than those fed ESBM100. Fecal scores were similar among animals fed the five diets during pre- and post-weaning and the entire period of the experiment (Table 5).

### Blood metabolites

Pre-weaning, serum glucose concentration tended to increase linearly as the level of ESBM in the starter increased (Table 6; p = 0.07). Serum urea concentration followed a quadratic pattern with incrasing ESBM level in starter (p< 0.05). Animals fed ESBM100 had a greter blood urea than those fed ESBM50 (p<0.05), with those fed the others diets intermediate. Blood BHBA increased linearly as the level of ESBM in the starter increased (p<0.05). Post-weaning, blood metabolites were similar among animals fed the five diets (Table 6).

Over the whole period, serum glucose (p<0.01) and blood BHBA (p = 0.05) increased linearly as the level of ESBM in the starter increased. Serum urea tended to follow a quadratic mode as the level of ESBM in the starter increased, with the lowest serum urea concentration in calves fed ESBM50 (Table 6; p = 0.07). Concentration of serum total protein tended to decrease with increasing ESBM level in the starter (p = 0.07).

## DISCUSSION

### Protein molecular weight distribution

Results of the present study indicate that protein of SBM is broken down into the smaller protein fractions during extrusion processing as indicated by SDS-PAGE analysis. Th results are in consistent with those reported by Sadeghi et al [2] in the case of disappearance of SBM protein bands after processing by autoclaving. All the four SDS-PAGE bands largely disappeared in ESBM. This suggests that larger size proteins were broken down into smaller sized protein, likely because of disruption of hydrogen bands within protein internal structures [1,2].

### Fourier transform infrared spectroscopy molecular structures

Extrusion of SBM increased the FTIR ratio of amide I:amide II height and also FTIR protein region, amide II region and non-structural carbohydrates region of ESBM were fully distinguished from SBM by PCA analysis, which may indicate changes in protein structural make-up. The protein primary structural bands including peak height and peak area of amide I and amide II indicate quantitative differences in protein functional groups, whereas their ratios indicate variation in protein structural make-up [1]. Current findings are consistent with the results of Allan and Booth [20] and Ruiz-Ruiz et al [21] who reported that changes in secondary, tertiary and quaternary protein structure occur as a consequence of high temperature extrusion processing. Alpha-helix and beta-sheets did, however, not change after extrusion of SBM in the current study, which is different from findings of Samady and Yu [1] who reported that moist heating (autoclaving at 120°C for 1 h) of soybean seed increased amid I: amid II ratio and decreased alpha helix: beta sheet ratio. Samadi and Yu [1] reported that the sensitivity of soybean seeds samples to moist heating was much greater than that to dry heating in terms of the FTIR molecular structure and nutrient profile changes.

### Chemical composition and CNCPS protein fractions

In current study, ESBM contained less CP and greater EE content compared with SBM. This makes sense because approximately 20 g/kg of saturated fat powder (commercially available as RP10, IFFCO, Johor, Malaysia) is used as a lubricant material during extrusion to reduce retention time through extruder and to avoid thermal damage of the SBM. In the study of Doiron et al [22] and Samadi and Yu [1], moist heating increased NDICP fraction of CP whereas in the current study this fraction remained stable after extrusion. In line with the results of Samadi and Yu [1] for soybean seed and Doiron et al [22] for flaxseed, moist heating did not change the un-degradable fraction (PC) of protein, also this fraction did not change in current samples. It has been widely accepted that the Maillard reactions will form when soybean seeds are being processed or cooked at high temperature [23]. Controlling this reaction by optimizing the heating process is the key to successful protection of soybean seed and SBM protein [5]. The CNCPS PC fraction of protein may increase when Maillard reactions have occurred during heat processing [23].

### Calves performance and nutrient digestibility

Over the whole experiment, weekly body weight, DMI, ADG and final body weight increased linearly as the level of ESBM in the starter increased, while the FCR remained stable. The effect of processing method on protein quality of SBM might be more important and easy to detect in young dairy calves than in older calves because of their less developed gastrointestinal tract [4]. Among different protein sources, calves preferred SBM to other feeds in short-term preference tests [4], suggesting that SBM is a palatable feed for calves. Processing of soybean seed alters flavor, color, texture, and other functional properties of proteins [3]. Therefore, a reason for the increased starter DMI and subsequently increased ADG and body weight might be due to increased palatability or decreased anti-nutritional factors for starter feed with ESBM [3,24].

Processing of soybean seed alters functional properties of its proteins, with concequent changes protein digestibility [3]. In the current study, results from SDS-PAGE indicated that the major bands of protein narrowed, indicating that big proteins were broken down into small proteins such as peptides [7], which may have greater digestibility. Current results indicated a linear increase in nutrient digestibility with increasing ESBM level in diet increased, altough this was mainly apparent at post-weaning. Moisture in combination with heating may either decrease or increase the digestion of protein in the gastrointestinal tract, which depends on the heating temperature, time of heating and amount of moisture. Allan and Booth [20] and Ruiz-Ruiz et al [21] demonstrated that during high temperature extrusion cooking, the stability of protein structures decreased, and as a concequence polypeptides and peptides would be more available and more hydrolysable by digestive enzymes. On the other hand, over processing would result in denaturation of protein and probably transform the proteins to a more resistant structure and formation of cross-linkages between amino acids and reducing sugars (i.e., Maillard reaction) could occur [23]. The un-digestible protein C fraction did, however, not change with extrusion suggesting that Maillard reaction did not form.

The improved performance of dairy calves fed starter feed with increasing levels of ESBM might be related to changes in CNCPS A and PB1 protein fractions, changes in FTIR protein and amide II region and amide I:amide II ratio and decreased electrophoretic protein molecular size. Changes in protein fractions, molecular structural make-up and molecular weights might lead to changes in rumen degradable protein (RDP) and rumen un-degradable protein (RUP) fractions in adult ruminant [1,2]. In the study of Samady and Yu [1] autoclave heating of soybean seed at 120°C increased intestinal digestibility of RUP. A reduction in rapidly degradable fraction of protein (PA) with consequent increase in PB1 fraction found in the current study due to extrusion could increase the RUP fraction of CP [23]. Previously, however, increasing the RUP fraction in dairy calves starter resulted in a improved performance response [25], a negative response [26] or no response [27]. Feeding heat-treated corn as an energy source and extruded whole soybean seed as a protein source to calves improved their efficiency of energy and nitrogen utilization for growth [28]. Kazemi-Bonchenari et al [8] demonstrated that a increasing RUP concentration in the starter feed by replacing half of SBM with xylose treated SBM improved feed efficiency in dariy calves through decreased DMI while body weight changes remained stable. On the other hand, Kazemi-Bonchenari et al [9] reported that increasing RUP content of starter feed had not beneficial effects in terms of feed intake or feed efficiency. Kazemi-Bonchenari et al [9] demonstrated that providing RUP with soy proteins compared to RDP from the same source might decrease ruminal microbial protein synthesis in pre-weaned calves. More researches with other sources of RUP might be required to determine if RUP with different amino acids (AA) compositions could increase calf performance or not. The FTIR spectra in the total carbohydrates and non-structural carbohydrates region differed between ESBM and SBM. These changes in carbohydrate molecular structures of ESBM might explain increased digestibility. This suggests that changes in protein quality due to extrusion of SBM should not be considered as the sole reason for improving the performance of dairy calves fed ESBM.

### Body size measurement

Body height and heart girth of dairy calves increased linearly pre-weaning, post-weaning and throughout the study as the level of ESBM in the starter increased. The increased structural growth of calves fed increasing levels of ESBM might be the result of additional energy and nutrients available for skeletal deposition due to the observed increase in DMI and CP digestibility. Moallem et al [29] found that increasing RUP content of the post-weaning diet of calves could accelerate skeletal growth rate. Kazemi-Bonchenari et al [8] reported that replacing 50% of SBM with xylose treated SBM did not change body size measurements such as body length, height and hearth girth in dairy calves. In another study, Kazemi-Bonchenari et al [9] did not find an effect of feeding a starter with higher RUP (43.0 vs 68.0 g/kg DM) on body measurements.

Feeding ESBM50 decreased heart girth of dairy calves in current study. This was consistent with a reduction in CP digestibility and blood urea nitrogen (BUN) pre-weaning at this level of ESBM. This may indicate that current ESBM level had decreased protein accessibility for microbial population in the rumen, therefore reduced rumen function and performance and consequently its development.

### Blood metabolites

The BUN decreased in calves fed with ESBM50 starter during pre-weaning. This may be partially due to the reduction in starter intake and CP digestibility compared with calves fed with ESBM100 starter. Kazemi-Bonchenari et al [8] found that replacing SBM with xylose treated SBM decreased plasma urea of calves while Kazemi-Bonchenari et al [9] found that feeding starter with a greater RUP content (68.0 vs 43.0 gr/kg DM) did not influence BUN concentration. Pre-weaning, and also over the whole experimental period, blood glucose and BHBA increased as the level of ESBM in the starter increased. The greater BHBA level in blood may reflect a more developed rumen. Butyrate has been found to stimulant papillae growth and is a primary energy source for rumen epithelium which when oxidised is converted to BHBA in ruminal wall [23]. Glucose and BHBA could be used as energy sources for some body peripheral tissues in calves and promote the growth of calves [23].

## CONCLUSION

Findings of the current study indicate that extrusion of SBM changed FTIR protein region area and amide I: amide II ratio, decreased molecular size of protein and changed CNCPS PA and PB1 fractions. Including ESBM in starter feed of female Holestein calves increased DMI, blood glucose and BHBA, skeletal growth and body weight. Dairy calves performance and physiological responses during pre and post-weaning were sensitive to SBM protein characteristics including electrophoretic size, FTIR molecular strcutures and CNCPS sub-fractions.

## Figures and Tables

**Table 1:** Ingredients and chemical composition of experimental starter feeds (% DM)

Items	Diets^1)^				

	SBM	ESBM25	ESBM50	ESBM75	ESBM100
Corn grain (fine ground)	42.2	42.2	42.2	42.2	42.2
Soybean meal	32.4	24.2	16.2	8.1	0.0
Extruded SBM	0.0	8.2	16.3	24.3	32.4
Barley	14.6	14.6	14.6	14.6	14.6
Wheat bran	4.2	4.2	4.2	4.2	4.2
Chopped alfalfa hay	2.4	2.4	2.4	2.4	2.4
Mineral supplement^2)^	1.0	1.0	1.0	1.0	1.0
Vitamin supplement^3)^	1.0	1.0	1.0	1.0	1.0
DCP	0.6	0.6	0.6	0.6	0.6
Salt	0.6	0.6	0.6	0.6	0.6
Na- bicarbonate	1.0	1.0	1.0	1.0	1.0
Chemical composition (% DM)					
CP	23.3	23.1	23.0	22.8	22.6
ADF	7.4	7.3	7.3	7.3	7.2
aNDF	15.5	15.7	16.0	16.3	16.6
CA	8.8	8.9	8.9	9.0	9.0
EE	2.7	2.8	2.9	3.0	3.1
NFC^4)^	49.7	49.5	49.3	49.0	48.8
Starch	42.1	42.0	41.8	41.6	41.5
Predicted energy contents (kcal/kg DM)					
ME	2,790	2,790	2,800	2,800	2,810
NEg	1,220	1,230	1,230	1,230	1,240

DM, dry matter; DCP, di-calcium phosphate; CP, crude protein; ADF, acid detergent fiber; aNDF, neutral detergent fiber analyzed with heat stable α-amylase; CA, crude ash; EE, ether extract; NFC, non-fiber carbohydrates; ME, metabolizable energy; NEg, net energy for gain.

1) SBM, diet based on soybean meal; ESBM25, 25% of soybean meal replaced by extruded soybean meal; ESBM50, 50% of soybean meal replaced by extruded soybean meal; ESBM75, 75% of soybean meal replaced by extruded soybean meal; ESBM100, 100% of soybean meal replaced by extruded soybean meal.

2) Contained per kilogram of supplement: 250,000 IU of vitamin A, 50,000 IU of vitamin D, 1,500 IU of vitamin E.

3) Contained per kilogram of supplement, 2.25 g of Mn, 120 g of Ca, 7.7 g of Zn, 20 g of P, 20.5 g of Mg, 186 g of Na, 1.25 g of Fe, 3 g of S, 14 mg of Co, 1.25 g of Cu, 56 mg of I, and 10 mg of Se.

4) Calculated as 100–(CP+EE+aNDF+CA).

**Table 2:** Chemical composition, protein fractions and FTIR protein molecular structures of soybean meal and extruded soybean meal

Items	SBM	ESBM	SEM	p-value
Chemical composition (% DM)				
CP	55.8	53.4	0.65	0.07
EE	0.8	2.1	0.26	0.03
ADF	9.5	9.0	0.45	0.39
CA	7.8	8.3	1.30	0.72
aNDF	16.6	20.5	1.31	0.11
Crude protein fractions^1)^ (% CP)				
A	13.5	7.5	0.56	<0.01
B1	1.2	2.4	0.07	<0.01
B2	61.0	60.7	5.09	0.94
B3	14.2	18.7	4.23	0.40
C	10.1	10.8	1.21	0.60
FTIR protein molecular structures^2)^				
Amide I	0.0397	0.0447	0.0039	0.38
Amide II	0.0257	0.0200	0.0025	0.14
Amide I:amide II	1.5706	2.2499	0.1364	<0.01
Alpha helix	0.0655	0.0498	0.0336	0.75
Beta sheets	0.4315	0.3366	0.1544	0.67
Alpha helix:beta sheets	0.2035	1.1224	0.7391	0.40
Others	0.5031	0.6136	0.1663	0.65

FTIR, fourier transform infrared spectroscopy; SBM, soybean meal; ESBM, extruded soybean meal; SEM, standard error of means; CP, crude protein; EE, ether extract; ADF, acid detergent fiber; CA, crude ash; aNDF, neutral detergent fiber analyzed with heat stable α-amylase.

1) Protein fractions according to CNCPS include PA, fraction of CP that is instantaneously solubilized at time zero and determined as NPN; PB1, soluble true protein calculated as buffer soluble CP minus non-protein nitrogen; PB2, intermediate degradable true protein calculated as CP – (PA + PB1 + PB3 + PC); PB3, slowly degradable true protein calculated as NDICP – ADICP; PC, undegradable CP determined as ADICP.

2) The FTIR structures were amide I area (ca. 1,720 to 1,575 cm^−1^), amide II area (ca. 1,575 to 1,485 cm^−1^) and ratio of amide I to amide II; for the α-helix and β-sheets, the peak falls within the range of ca. 1,650 to 1,660 and 1,620 to 1,640 cm^−1^, respectively.

**Table 3:** Effect of substitution of soybean meal with extruded soybean meal in starter feed on performance of Holstein dairy calves during pre and post-weaning

Items	Treatments^1)^					SEM	Level of significant (p-value)			Response curves		
		
	SBM	ESBM25	ESBM50	ESBM75	ESBM100		Treat	Week	Treat×week	Linear	Quadratic	Qubic
Pre-weaning (kg)												
iBW	39.8	39.6	39.0	39.8	42.8	1.41	0.16	-	-	0.68	0.72	0.78
WW	78.6	77.7	79.4	80.9	83.7	1.9861	0.27	-	-	0.04	0.33	0.84
wBW	58.2^bc^	57.1^c^	58.2^bc^	59.0^b^	60.7^a^	0.48	<0.01	<0.01	0.99	<0.01	<0.01	0.18
SI (g/d)	379.2	371.5	364.6	321.8	407.6	28.57	0.054	<0.01	0.85	0.91	0.05	0.05
tDMI (g/d)	981.9	974.3	967.4	924.5	1,010.3	28.57	0.054	<0.01	0.85	0.91	0.05	0.05
ADG (g/d)	526.9	506.2	540.0	546.6	568.7	29.85	0.30	<0.01	0.82	0.06	0.46	0.55
FCR	2.4	2.4	2.8	2.4	2.7	0.55	0.92	0.06	0.95	0.74	0.84	0.82
Post weaning												
BW (kg)	87.0^b^	85.7^b^	90.7^ab^	91.2^ab^	96.3^a^	2.18	<0.01	<0.01	<0.01	<0.01	0.15	0.71
DMI (g/d)	1,885.5	1,790.0	2,074.0	1,998.1	2,241.0	158.16	0.06	<0.01	0.95	<0.05	0.45	0.86
ADG (g/d)	694.3^ab^	645.5^b^	820.6^ab^	781.4^ab^	925.9^a^	70.37	0.05	0.14	0.84	<0.01	0.51	0.85
FCR	3.4	3.7	3.8	3.2	3.2	1.11	0.10	0.02	0.95	0.25	0.28	0.05
Entire period												
wBW (kg)	64.4^bc^	63.2^c^	65.1^b^	65.9^b^	68.3^a^	0.62	<0.01	<0.01	<0.01	<0.01	<0.01	0.21
DMI (g/d)	1,175.5^ab^	1,149.1^b^	1204.5^ab^	1,154.6^ab^	1,274.1^a^	41.56	0.02	<0.01	0.85	0.03	0.09	0.34
ADG (g/d)	564.0^ab^	536.9^b^	599.4^ab^	596.8^ab^	643.2^a^	30.92	0.01	<0.01	0.85	<0.01	0.32	0.55
FCR	2.6	3.3	3.0	2.6	2.8	0.54	0.62	<0.01	0.85	0.69	0.53	0.18
fBW (kg)	90.8	89.7	93.5	95.3	100.0	3.03	0.16	-	-	0.02	0.48	0.72

SEM, standard error of means; iBW, initial body weight; SI, starter intake; tDMI, total dry matter intake, FCR, feed conversion ratio; ADG, average daily gain; BW, body weight; fBW, final body weight.

1) SBM, diet based on soybean meal; ESBM25, 25% of soybean meal replaced by extruded soybean meal; ESBM50, 50% of soybean meal replaced by extruded soybean meal; ESBM75, 75% of soybean meal replaced by extruded soybean meal; ESBM100, 100% of soybean meal replaced by extruded soybean meal.

a–c Means with different letters within the same row differ (p<0.05).

**Table 4:** Effect of substitution of soybean meal with extruded soybean meal in starter feed on nutrient digestibility in Holstein dairy calves during pre and post-weaning

Items	Treatments^1)^					SEM	Level of significance			
	
	SBM	ESBM25	ESBM50	ESBM75	ESBM100		Treat	Linear	Quadratic	Cubic
Week 4 (%)										
DMD	57.4	66.4	61.5	57.6	66.2	5.04	0.23	0.44	0.98	0.03
OMD	59.3	68.0	60.1	58.5	67.0	5.02	0.23	0.61	0.73	0.03
CPD	74.2^b^	87.2^a^	74.1^b^	79.6^ab^	83.8^a^	3.32	0.04	0.28	0.93	0.02
Week 10 (%)										
DMD	63.4	61.5	68.3	67.4	62.8	3.05	0.13	0.47	0.11	0.08
OMD	65.0	63.5	70.0	68.9	63.9	2.32	0.20	0.67	0.11	0.12
CPD	71.0	70.2	75.9	78.2	72.3	2.53	0.16	0.19	0.16	0.08
Week 13 (%)										
DMD	56.2	57.9	56.0	62.3	64.1	2.92	0.21	0.04	0.45	0.92
OMD	58.0	59.9	57.1	63.9	65.7	3.03	0.23	0.06	0.41	0.97
CPD	77.2^b^	78.7^ab^	72.6^b^	81.0^ab^	83.7^a^	2.34	0.03	0.05	0.06	0.45

SEM, standard error of means; DMD, dry matter digestibility; OMD, organic matter digestibility; CPD, crude protein digestibility.

1) SBM, diet based on soybean meal; ESBM25, 25% of soybean meal replaced by extruded soybean meal; ESBM50, 50% of soybean meal replaced by extruded soybean meal; ESBM75, 75% of soybean meal replaced by extruded soybean meal; ESBM100, 100% of soybean meal replaced by extruded soybean meal.

ab Means with different letters within the same row differ (p<0.05).

**Table 5:** Effect of substitution of soybean meal with extruded soybean meal in starter feed on Holstein dairy calves body size measurements and fecal score during pre and post-weaning

Items	Treatments^1)^					SEM	Level of significant (p-value)			Response curves		
		
	SBM	ESBM25	ESBM50	ESBM75	ESBM100		Treat	Week	Treat×week	Linear	Quadratic	Qubic
Pre-weaning												
IHeight^2)^ (cm)	79.1	78.6	78.1	78.8	79.7	0.94	0.80	-	-	0.63	0.25	0.94
iHeart girth^2)^ (cm)	77.9	78.3	77.6	78.7	79.5	1.00	0.70	-	-	0.25	0.48	0.70
Height (cm)	86.2^ab^	85.4^b^	86.3^ab^	86.4^ab^	87.2^a^	0.53	0.03	<0.01	0.95	0.01	0.13	0.42
Heart girth (cm)	88.5^b^	87.7^bc^	87.5^c^	89.1^ab^	89.7^a^	0.31	<0.01	<0.01	0.85	<0.01	<0.01	0.02
Fecal score	3.0	3.1	3.1	3.1	3.1	0.069	0.94	<0.01	0.75	0.47	0.64	0.96
Post-weaning												
Height (cm)	95.9^b^	95.8^b^	96.0^ab^	96.6^ab^	97.8^a^	0.65	0.02	<0.01	0.92	<0.01	0.08	0.45
Heart girth (cm)	100.3^ab^	99.1^b^	100.3^ab^	101.0^ab^	102.3^a^	0.74	<0.01	<0.01	0.84	<0.01	0.02	0.24
Fecal score	3.4	3.3	3.3	3.4	3.4	0.13	0.51	0.10	0.69	0.65	0.13	0.37
Entire period												
Height (cm)	88.3^ab^	87.6^b^	88.4^ab^	88.6^ab^	89.5^a^	0.31	<0.01	<0.01	0.88	<0.01	0.04	0.49
Heart girth (cm)	91.0^bc^	90.1^c^	90.2^c^	91.6^ab^	92.4^a^	0.21	<0.01	<0.01	0.75	<0.01	<0.01	<0.01
Fecal score	3.2	3.2	3.1	3.2	3.1	0.06	0.93	<0.01	0.87	0.41	0.92	0.78

SEM, standard error of means.

1) SBM, diet based on soybean meal; ESBM25, 25 percent of soybean meal replaced by extruded soybean meal; ESBM50, 50 percent of soybean meal replaced by extruded soybean meal; ESBM75, 75 percent of soybean meal replaced by extruded soybean meal; ESBM100, 100 percent of soybean meal replaced by extruded soybean meal.

2) iHeight, withers height of calves at the beginning of experiment; iHeart girth, heart girth of calves at the beginning of experiment.

a–c Means with different letters within the same row differ (p<0.05).

**Table 6:** Effect of substitution of soybean meal with extruded soybean meal in starter feed on blood metabolites of Holstein dairy calves during pre and post-weaning

Items	Treatments^1)^					SEM	Level of significant (p-value)			Response curves		
		
	SBM	ESBM25	ESBM50	ESBM75	ESBM100		Treat	Week	Treat×week	Linear	Quadratic	Qubic
Pre-weaning period												
Glucose (mg/dL)	92.8	98.0	104.3	106.3	106.2	5.98	0.43	<0.01	0.75	0.07	0.50	0.87
Urea (mg/dL)	21.6^ab^	19.0^ab^	16.6^b^	20.8^ab^	22.0^a^	1.26	0.02	0.04	0.68	0.49	<0.01	0.44
Albumin (g/dL)	3.1	3.0	2.9	3.1	3.0	0.05	0.11	0.32	0.85	0.95	0.28	0.26
Total protein (g/dL)	6.4	6.5	6.3	6.1	6.2	0.21	0.61	<0.01	0.92	0.15	0.97	0.48
Globulin (g/dL)	3.4	3.4	4.0	3.0	3.1	0.28	0.12	<0.01	0.69	0.27	0.20	0.44
BHBA (mg/dL)	0.25	0.25	0.27	0.28	0.32	0.026	0.3181	<0.01	0.85	0.04	0.58	0.81
Post-weaning												
Glucose (mg/dL)	65.8	60.3	64.5	65.8	71.5	6.44	0.80	-	-	0.41	0.43	0.81
Urea (mg/dL)	25.1	27.2	29.0	25.6	30.0	3.09	0.76	-	-	0.41	0.95	0.42
Albumin (g/dL)	3.1	3.1	3.1	3.2	3.0	0.07	0.51	-	-	0.86	0.39	0.24
Total protein (g/dL)	6.3	6.6	6.0	6.3	6.1	0.22	0.36	-	-	0.26	0.93	0.60
Globulin (g/dL)	3.2	3.5	2.9	3.1	3.1	0.22	0.44	-	-	0.29	0.86	0.38
BHBA (mg/dL)	0.38	0.35	0.48	0.48	0.40	0.096	0.83	-	-	0.57	0.54	0.47
Entire period												
Glucose (mg/dL)	83.4	85.5	91.2	93.0	94.6	6.49	0.13	<0.01	0.77	<0.01	0.76	0.84
Urea (mg/dL)	22.7	21.8	20.7	22.4	24.7	1.33	0.32	<0.01	0.65	0.28	0.07	0.88
Albumin (g/dL)	3.1	3.1	3.0	3.1	3.0	0.04	0.06	0.06	0.95	0.88	0.68	0.11
Total protein (g/dL)	6.4	6.5	6.2	6.1	6.1	0.16	0.36	<0.01	0.82	0.07	0.97	0.40
Globulin (g/dL)	3.3	3.6	3.6	3.0	3.1	0.20	0.20	<0.01	0.66	0.17	0.27	0.31
BHBA (mg/dL)	0.28	0.28	0.32	0.33	0.34	0.028	0.38	<0.01	0.49	0.05	0.96	0.72

SEM, standard error of means.

1) SBM, diet based on soybean meal; ESBM25, 25 percent of soybean meal replaced by extruded soybean meal; ESBM50, 50 percent of soybean meal replaced by extruded soybean meal; ESBM75, 75 percent of soybean meal replaced by extruded soybean meal; ESBM100, 100 percent of soybean meal replaced by extruded soybean meal.

ab Mans with different letters within the same row differ (p<0.05).
